# Impaired CD4^+^ T cell response in older adults is associated with reduced immunogenicity and reactogenicity of mRNA COVID-19 vaccination

**DOI:** 10.1038/s43587-022-00343-4

**Published:** 2023-01-12

**Authors:** Norihide Jo, Yu Hidaka, Osamu Kikuchi, Masaru Fukahori, Takeshi Sawada, Masahiko Aoki, Masaki Yamamoto, Miki Nagao, Satoshi Morita, Takako E. Nakajima, Manabu Muto, Yoko Hamazaki

**Affiliations:** 1 Department of Life Science Frontiers, Center for iPS Cell Research and Application (CiRA), Kyoto University, Kyoto, Japan; 2 Alliance Laboratory for Advanced Medical Research, Graduate school of Medicine, Kyoto University, Kyoto, Japan; 3 Department of Biomedical Statistics and Bioinformatics, Graduate School of Medicine, Kyoto University, Kyoto, Japan; 4 Department of Therapeutic Oncology, Graduate School of Medicine, Kyoto University, Kyoto, Japan; 5 Clinical Bio-Resource Center, Kyoto University Hospital, Kyoto, Japan; 6 Department of Early Clinical Development, Graduate school of Medicine, Kyoto University, Kyoto, Japan; 7 Kyoto Innovation Center for Next Generation Clinical Trials and iPS Cell Therapy (Ki-CONNECT), Kyoto University Hospital, Kyoto, Japan; 8 Department of Clinical Laboratory Medicine, Graduate School of Medicine, Kyoto University, Kyoto, Japan; 9 Laboratory of Immunobiology, Graduate school of Medicine, Kyoto University, Kyoto, Japan

**Keywords:** Lymphocytes, Ageing, Infection

## Abstract

Whether age-associated defects in T cells impact the immunogenicity and reactogenicity of mRNA vaccines remains unclear. Using a vaccinated cohort (*n* = 216), we demonstrated that older adults (aged ≥65 years) had fewer vaccine-induced spike-specific CD4^+^ T cells including CXCR3^+^ circulating follicular helper T cells and the T_H_1 subset of helper T cells after the first dose, which correlated with their lower peak IgG levels and fewer systemic adverse effects after the second dose, compared with younger adults. Moreover, spike-specific T_H_1 cells in older adults expressed higher levels of programmed cell death protein 1, a negative regulator of T cell activation, which was associated with low spike-specific CD8^+^ T cell responses. Thus, an inefficient CD4^+^ T cell response after the first dose may reduce the production of helper T cytokines, even after the second dose, thereby lowering humoral and cellular immunity and reducing systemic reactogenicity. Therefore, enhancing CD4^+^ T cell response following the first dose is key to improving vaccine efficacy in older adults.

## Abstract (web summary)

Impaired CD4^+^ T cell responses in older adults correlate with weaker humoral and cellular immunity as well as reduced systemic reactogenicity following mRNA coronavirus disease 2019 vaccination, thus highlighting the impact of T cell aging on vaccine effectiveness.

## Main

Advanced age is the most important risk factor for severe coronavirus disease 2019 (COVID-19) outcomes^1–3^; this may be largely due to the age-associated decline in immune competence. T cells are immune cells that belong to the adaptive immune system and play a central role in antigen-specific antibody response and cytotoxicity against virus-infected cells^4^. Despite their critical roles, the production of new T cells begins to decline during early life stages due to thymic involution and undergoes various qualitative and compositional changes and functional dysregulations with age^5–11^. Thus, older individuals are strongly recommended to receive vaccines; however, the benefits and efficacy of vaccination are limited, primarily due to the decreased effectiveness of adaptive immunity^12–14^.

The newly developed severe acute respiratory syndrome coronavirus 2 (SARS-CoV-2) mRNA vaccines are highly effective at preventing severe illness, as well as infection, at ~95% efficacy, even in participants aged ≥65 years^15^. However, spike-specific IgG levels and neutralizing antibody titers are significantly lower in older individuals^16–19^. Detailed immunological studies revealed that the mRNA vaccines elicit strong type 1 helper T (T_H_)1) cell and follicular helper T (T_FH_) cell responses^20,21^. Importantly, older adults, especially individuals >80 years of age, showed fewer cytokine-positive CD4^+^ T cells after vaccination^16,19^. However, the detailed trajectory of T cell responses and how T_H_1 and T_FH_ cell responses are affected in older adults remains to be investigated. Considering the importance of T cells in vaccine responses, elucidating age-associated differences in T cell responses to mRNA vaccines is fundamental.

Noticeable and severe adverse effects (AEs) are another characteristic of mRNA vaccines^22^. Notably, AEs are more frequent and more severe after the second dose^15^, which strongly suggests that AEs are a consequence of immunological memory. However, previous reports did not reach a consensus concerning the association between AEs and vaccine-induced immune reactions, likely due to the small cohorts and variations of the definition of AEs^23–27^. Moreover, most studies have examined the associations of AEs with the humoral immune response but not with T cell responses, which can result in the production of cytokines and thus cause systemic effects.

In this study, we investigated these key questions by comparing the spike-specific T_H_1 cell and T_FH_ cell responses to two doses of mRNA vaccine between adults and older adults in a Japanese cohort of healthy individuals over 3 months after vaccination, including the priming and contraction phases. Furthermore, we explored the associations of spike-specific T cell responses with AEs. Our results provide an improved understanding of the mechanisms of age-related and individual differences in the effectiveness of mRNA vaccines and may be relevant for future vaccine strategies, especially for the highly vulnerable older population.

## Results

### Lower induction and early contraction of CD4^+^ T cell responses in older adults

**Extended Data Fig. 1:**
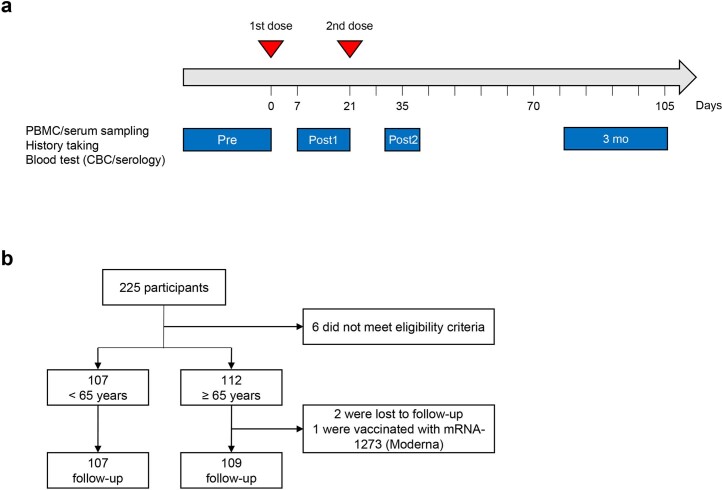
Study design. (**a**) Participants received the first BNT162b2 dose on day 0 and second on around day 21. The sampling points were set with an allowance: 7–21 days after the first dose (Post1), 31–39 days after the first dose (Post2), and 79–104 days at the point of 3 months after the first dose (3 mo). The actual vaccinated and sampling days are described in the Results section. CBC; complete blood count. (**b**) The diagram represents all participants throughout the study.

We studied 216 SARS-CoV-2-naïve Japanese donors comprising adults (aged <65 years; median age, 43 years; *n* = 107) and older adults (aged ≥ 65 years; median age, 71 years; *n* = 109) who met the eligibility criteria (see ‘Study design’ in the Methods), having received two doses of BNT162b2 vaccine within around 3-week intervals (median, 21.0 d; range, 19.0 to 30.0 d), and were successfully followed up until 3 months after the first dose (Extended Data Fig. 1a,b and Table 1). Blood samples were obtained before the vaccination (Pre; median, −14 d (range, −29 to 0 d)), 2 weeks after the first dose (Post1; median, 11 d (range, 6–21 d)), 2 weeks after the second dose (Post2; median, 34 d (range, 30−39 d)) and 3 months after the first dose (3 mo; median, 93 d (range, 77–104 d; Extended Data Fig. 1a). Donors were also followed up for medical conditions at each study visit. None of the donors tested positive for anti-SARS-CoV-2 nucleocapsid (N) protein IgM/IgG, which reflects the history of COVID-19 at enrollment (Table 1).

**Table 1:** Participant characteristics at enrollment

			Adults (<65 years) *n* = 107	Older adults (≥65 years) *n* = 109
Age (years)	Median (range)		43 (23–63)	71 (65–81)
Sex	*n* (%)	Male	43 (40.2%)	56 (51.4%)
		Female	64 (59.8%)	53 (48.6%)
CMV IgG	*n* (%)	Negative	34 (31.8%)	9 (8.3%)
		Positive	73 (68.2%)	100 (91.7%)
SARS-CoV-2 N IgM/IgG	*n* (%)	Negative	107 (100%)	109 (100%)
		Positive	0 (0%)	0 (0%)

**Fig. 1:**
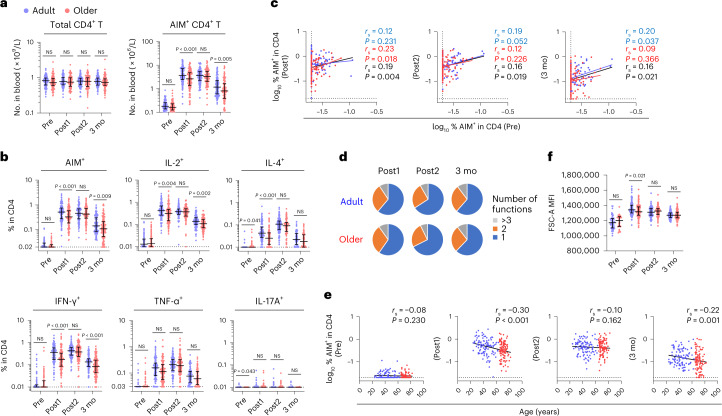
Lower induction and early contraction of spike-specific CD4^+^ T cells in older adults. **a**, Absolute number of total and AIM^+^ (CD137^+^CD154^+^) CD4^+^ T cells in blood. Pre, Post1, Post2 and 3 mo represents the sampling point before vaccination, after the first dose, after the second dose and 3 months after the first dose, respectively. **b**, Frequency of AIM^+^ and cytokine^+^ CD4^+^ T cells. **c**, Correlation between the percentage of AIM^+^ CD4^+^ T cells before and after vaccination. **d**, Proportions of multiple cytokine-expressing CD4^+^ T cells after vaccination in adult and older adult group. The blue, orange and gray colors in pie charts depict the production of one, two and more than three cytokines, respectively. **e**, Correlations between the percentages of AIM^+^ CD4^+^ T cells and age of donors. **f**, MFI of FSC-A in AIM^+^ CD4^+^ T cells. In **a**, **b** and **f**, the center line and error bars indicate the median and IQR, respectively. In **b**, **c** and **e**, the dotted line indicates limit of detection (LOD). Statistical comparisons across cohorts were performed using the Mann–Whitney test. Spearman’s rank correlation (*r*_s_) was used to identify relationships between two variables, with a straight line drawn by linear regression analysis. For correlation analysis, percentages of AIM^+^ CD4^+^ T cells were transformed into logarithmic values. NS, not significant. Blue, red and black characters represent the results of statistical test from adults (*n* = 107), older adults (*n* = 109) and both groups (*n* = 216), respectively. Source data

**Extended Data Fig. 2:**
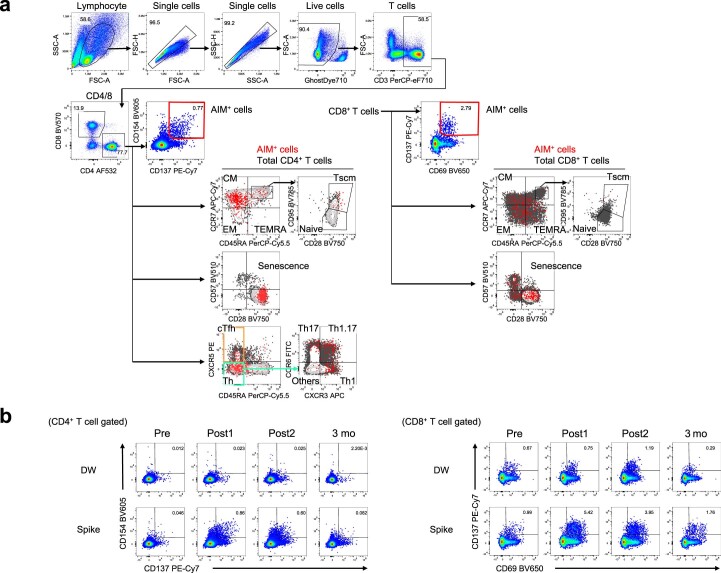
Identification and phenotyping of AIM^+^ T cells. (**a**) Representative gating strategy to detect and characterize spike-specific T cells in the AIM assay. Briefly, lymphocytes were gated out of all events, and doublets were excluded. Live cells were gated as Ghost Dye^TM^ Red 710^−^. T cells were then gated as CD3^+^ and subdivided into CD4^+^ and CD8^+^ populations. CD4^+^ and CD8^+^ T cells were further characterized for the phenotype such as differentiation, senescence, Th, and cTfh. Differentiation statuses were defined as naïve (Naïve, CD45RA^+^CCR7^+^CD28^+^CD95^−^), stem cell memory (Tscm, CD45RA^+^CCR7^+^CD28^+^CD95^+^), central memory (CM, CD45RA^−^CCR7^+^), effector memory (EM, CD45RA^−^CCR7^−^) or terminally differentiated effector memory cells re-expressing CD45RA (TEMRA, CD45RA^+^CCR7^−^). T cell senescence was assessed using CD28 and CD57. Th and cTfh were gated as CD45RA^−^CXCR5^−^ and CD45RA^−^CXCR5^+^, respectively, in CD3^+^CD4^+^ cells. Th was further divided into 1, 2, 17, and 1/17 subtypes based on the expression of CXCR3 and CCR6. Distributions of AIM^+^ cells in each phenotype are indicted in red dots. (**b**) Representative FCM plots displaying AIM^+^ (CD137^+^CD154^+^ for CD4, and CD69^+^CD137^+^ for CD8^+^) T cells after stimulation with the negative control (DW) or spike protein overlapping peptides (Spike). Numbers indicate the population percentages in the gates.

**Extended Data Fig. 3:**
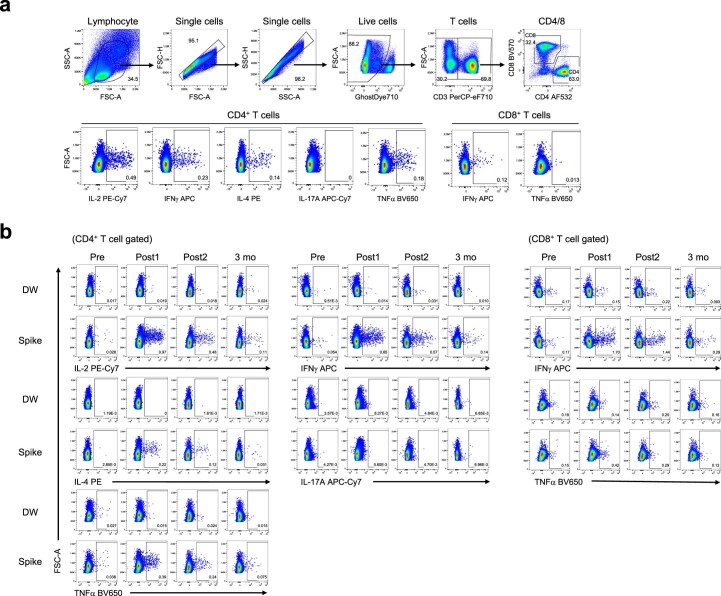
Identification of cytokine^+^ T cells. (**a**) Representative gating strategy to detect and characterize spike-specific T cells in the ICS assay. Briefly, lymphocytes were gated out of all events, and doublets were excluded. Live cells were gated as Ghost Dye^TM^ Red 710^−^. T cells were then gated as CD3^+^ and subdivided into CD4^+^ and CD8^+^ populations. CD4^+^ and CD8^+^ T cells were further analyzed for corresponding cytokines. (**b**) Representative FCM plots displaying cytokine^+^ CD4^+^ and CD8^+^ T cells after stimulation with the negative control (DW) or spike protein overlapping peptides (Spike). Numbers indicate the population percentages in the gates.

To quantify and characterize vaccine-induced T cell responses, we utilized activation-induced marker (AIM) and intracellular cytokine staining (ICS) assays (Extended Data Figs. 2 and 3)^20,21,28–32^. Peripheral blood mononuclear cells (PBMCs) were stimulated with overlapping peptide pools covering the complete sequence of the spike protein of SARS-CoV-2, which was used as a vaccine antigen. Markers used for flow cytometric analysis and the gating strategies are shown in Supplementary Table 1 and Extended Data Figs. 2 and 3. The total number of CD4^+^ T cells in peripheral blood did not differ between adults and older adults and remained stable during the study duration (Fig. 1a). The numbers and frequencies of spike-specific AIM^+^ (CD154^+^CD137^+^) CD4^+^ T cells in most donors exhibited a significant increase (median, >10-fold) as compared with the baseline after the first dose, were largely maintained after the second dose, and declined at 3 months (Fig. 1a,b), as previously reported^28–30^. However, older adults induced significantly fewer spike-specific CD4^+^ T cells than adults after the first dose (median (interquartile range; IQR), adults; 0.52% (0.47%) and older adults; 0.33% (0.40%) in total CD4^+^ T cells; *P* < 0.001), reached the same level as that of adults after the second dose, and again exhibited significantly lower levels at 3 months (Fig. 1a,b). The frequency of AIM^+^ CD4^+^ T cells before vaccination, which may include naïve as well as cross-reactive T cells^31,33^, showed a weak correlation with those after the first dose in older adults (*r*_s_ = 0.23, *P* = 0.018) (Fig. 1c), but not with those after the second dose, at 3 months (Fig. 1c), or peak antibody titers, suggesting a limited effect of preexisting T cells on immune responses to two doses of vaccination.

**Extended Data Fig. 4:**
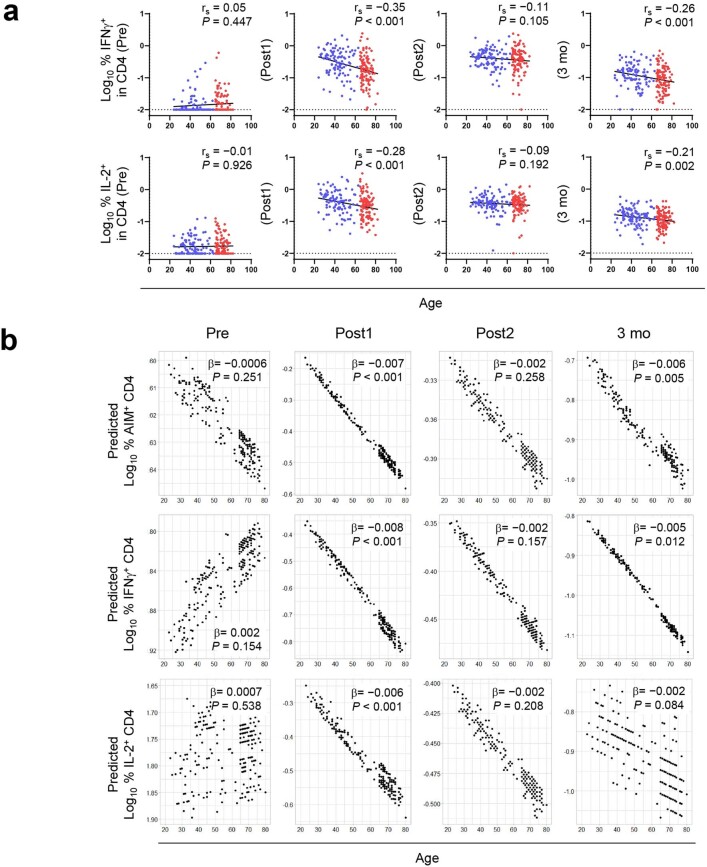
Correlations between the frequencies of spike-specific CD4^+^ T cells and age. (**a**) Correlation between the percentages of IFNγ^+^ or IL-2^+^ CD4^+^ T cells and donor age. Spearman’s rank correlation (r_s_) was used to identify relationships between two variables, with a straight line drawn by linear regression analysis. (**b**) Predicted percentages of AIM^+^, IFNγ^+^, and IL-2^+^ CD4^+^ T cells adjusted with days post vaccination was calculated using multiple regression analysis. Regression coefficients (β) of age and *P* values are shown. AIM^+^ and cytokine^+^ percentages were transformed into logarithmic values. Blue, red, and black dots represent adults (*n* = 107), older adults (*n* = 109), and both groups (*n* = 216), respectively. Source data

**Extended Data Fig. 5:**
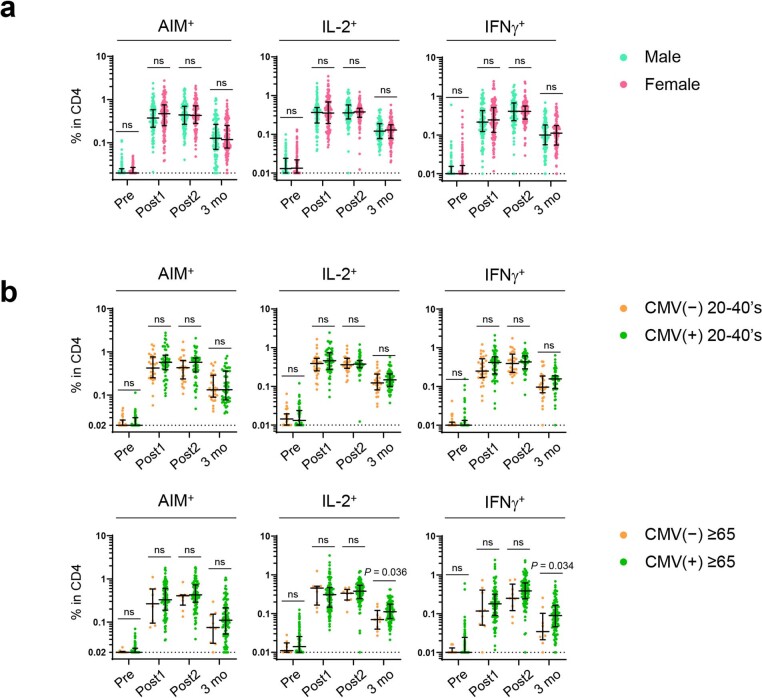
Comparison of spike-specific CD4^+^ T cells between male and female, and CMV-seronegative and seropositive. Frequency of AIM^+^ and cytokine^+^ CD4^+^ T cells from male (*n* = 99) and female (*n* = 117) donors (**a**) and CMV-seronegative and -seropositive donors aged 20–40 years (*n* = 29 and *n* = 54, respectively) (**b**, upper,) and in older adults (*n* = 9 and *n* = 100, respectively) (**b**, lower). The centerline and error bars indicate the median and IQR. The dotted line indicates limit of detection (LOD). Statistical comparisons across cohorts were performed using the Mann-Whitney U test. ns, not significant. Source data

Major cytokines induced in CD4^+^ T cells after vaccination were interleukin (IL)-2, interferon (IFN)-γ and tumor necrosis factor (TNF)-α, whereas IL-4^+^ and IL-17^+^ cells were fewer in frequency in both groups (Fig. 1b) as reported previously^20,29,30^. Boolean analysis indicated that two and multiple cytokine-producing cells were similarly observed following vaccination in both groups (Fig. 1d). However, the frequencies of cytokine-positive cells, especially IFN-γ^+^ cells, in older groups were significantly lower after the first dose and at 3 months than those in adults, similar to the kinetics of AIM^+^ cells (Fig. 1b). Indeed, the negative correlation between age as a continuous variable and AIM^+^ or cytokine-positive CD4^+^ T cells after the first dose and at 3 months, but not after the second dose, was observed (Fig. 1e and Extended Data Fig. 4a). To determine the effects of different sampling intervals following vaccination on T cell responses, multiple regression analysis was performed using the participant’s age and number of days after vaccination as explanatory variables and the predicted values of T cell responses were calculated. Regression coefficients (*β*) of age were negative after the first dose (AIM^+^, *β* = −0.007 and *P* < 0.001; and IFN-γ^+^, *β* = −0.008 and *P* < 0.001) and 3 months (AIM^+^, *β* = −0.006 and *P* = 0.005; and IFN-γ^+^, *β* = −0.005 and *P* = 0.012), confirming that T cell responses at both time points were negatively associated with age (Extended Data Fig. 4b). Previous studies have revealed that cytomegalovirus (CMV) infection and gender differences could affect vaccine responses^34,35^. No significant differences were found in the frequencies of AIM^+^ and cytokine-positive CD4^+^ T cells between males and females (Extended Data Fig. 5a) or CMV IgG-seropositive and IgG-seronegative individuals in the younger (20–40 years) or older (≥65 years) group (Extended Data Fig. 5b).

**Extended Data Fig. 6:**
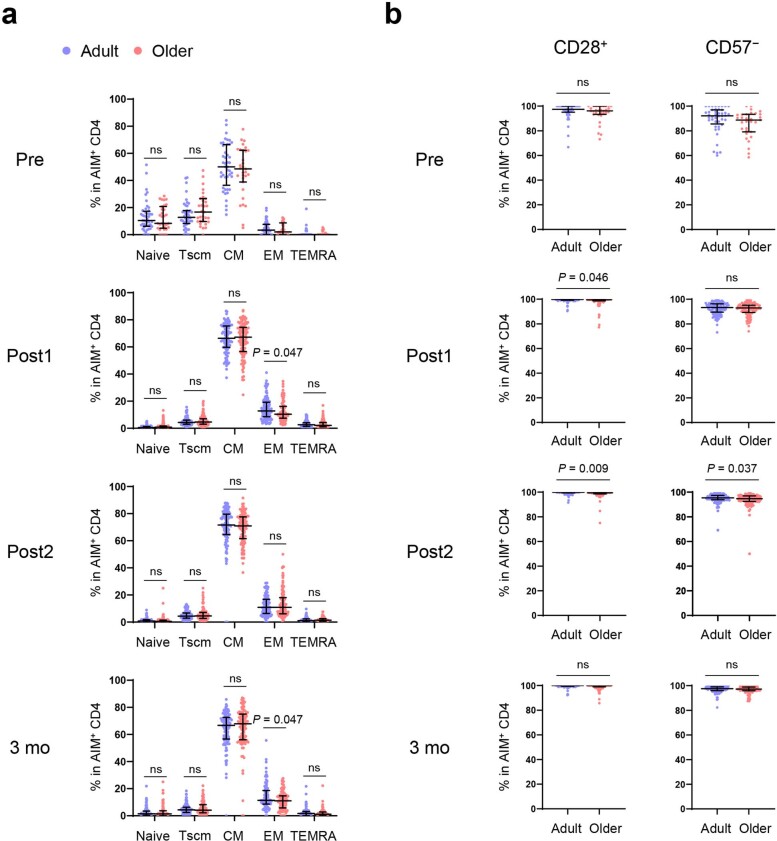
Differentiation and senescence status of spike-specific CD4^+^ T cells. (**a**) Frequency of Naïve, Tscm, CM, EM, and TEMRA in AIM^+^ CD4^+^ T cells. (**b**) Frequency of CD28^+^ and CD57^−^ in AIM^+^ CD4^+^ T cells. The centerline and error bars indicate the median and interquartile range (IQR). Statistical comparisons across cohorts were performed using the Mann-Whitney test. ns, not significant. Blue and red dots represent adults (*n* = 107) and older adults (*n* = 109), respectively. Source data

**Extended Data Fig. 7:**
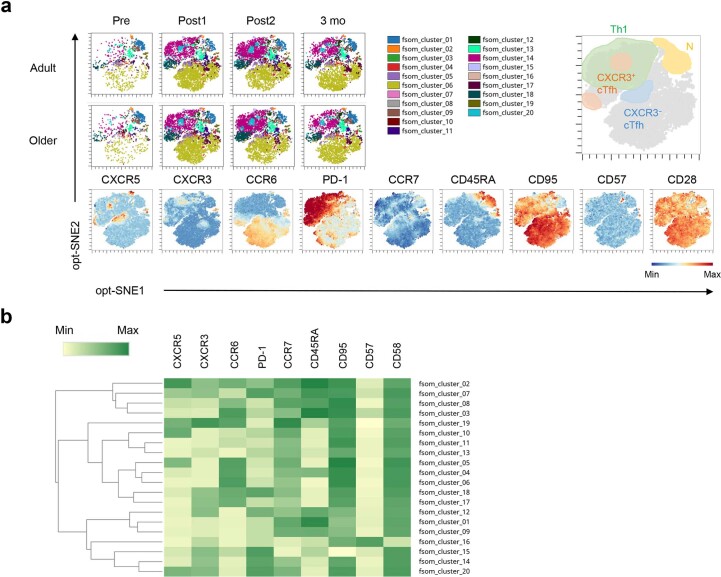
opt-SNE and FlowSOM for spike-specific CD4^+^ T cells. (**a**) FlowSOM clustering of AIM^+^ CD4^+^ T cells pooled from adults and older adults is displayed on opt-SNE plots. The markers described in the bottom row (CXCR5, CXCR3, CCR6, PD-1, CCR7, CD45RA, CD95, CD57, and CD28) were used for the analysis. AIM^+^ CD4^+^ cells were separated into 20 clusters. Th1(T_H_1), CXCR3^+^ cTfh, CXCR3^−^ cTfh, and N (naïve) cells were manually annotated based on the lineage marker expression. (**b**) Heatmap showing the marker expression for each FlowSOM cluster. Source data

**Extended Data Fig. 8:**
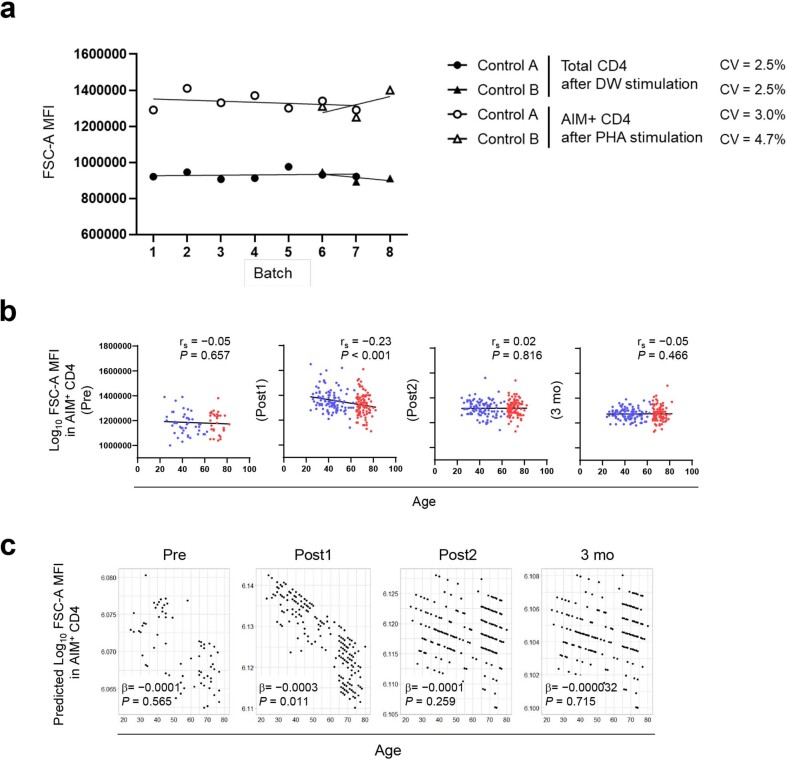
Correlation between the FSC-A MFI of spike-specific CD4^+^ T cells and age. (**a**) FSC-A MFI of total CD4^+^ T cells after DW stimulation and AIM^+^ CD4^+^ T cells after PHA stimulation in AIM assay. PBMC samples from the same lot (Sample A for exp #1–7 and Sample B for exp# 6–8) were included as internal quality control to define the variation inter-assay. Coefficient of variation (CV) are relatively stable across the eight independent experiments (exp #1–7; 3.0% with PHA and 2.5% with DW stimulation and exp# 6–8; 4.7% with PHA and 2.5 % with DW stimulation). (**b**) Correlations between FSC-A MFI in AIM^+^ CD4^+^ T cells and donor age. Spearman’s rank correlation (r_s_) was used to identify relationships between two variables, with a straight line drawn using linear regression analysis. (**c**) Predicted FSC-A MFI in AIM^+^ CD4^+^ T cells adjusted with days after vaccination was calculated using multiple regression analysis. Regression coefficients (β) of age and *P* values are shown. Blue, red, and black dots represent adults (*n* = 107), older adults (*n* = 109), and both groups (*n* = 216), respectively. Source data

Phenotypically, vaccine-induced T cells mostly present CCR7^+^CD45RA^−^ central memory (CM)^28^ and non-senescent (CD28^+^ or CD57^−^) characteristics after the first dose, which was maintained until 3 months in both groups (Extended Data Fig. 6a,b). Optimized *t*-distributed stochastic neighbor embedding (opt-SNE), multidimensional reduction strategy of multicolor flow cytometry data^36^ analysis also showed that spike-specific CD4^+^ T cells from all adult and older donors demonstrated similar fundamental characteristics (Extended Data Fig. 7). Notably, however, the cell size according to forward scatter (FSC) of flow cytometry, an indicator of T cell activation, peaked after the first dose in adults but after the second dose in the older group and decreased in both groups during the contraction phase at 3 months, whereas after the first dose the response in older adults was significantly lower than that in adults (Fig. 1f and Extended Data Fig. 8a). Consistently, the mean fluorescence intensity (MFI) of FSC-A was negatively correlated with age after the first dose (*r*_s_ = −0.23; *P* < 0.001; Extended Data Fig. 8b), while the regression coefficient (*β*) of age after the first dose was −0.0003 (*P* = 0.011) as determined via multiple regression analysis using the predicted FSC-A adjusted for days after vaccination (Extended Data Fig. 8c).

These results suggest that vaccine-induced spike-specific CD4^+^ T cells have similar characteristics in both groups, but that the older group exhibits lower induction and rapid contraction of antigen-specific CD4^+^ T cell responses after mRNA vaccination.

### Lower CXCR3^+^ T_FH_ cell induction is correlated with lower IgG levels in older adults

**Fig. 2:**
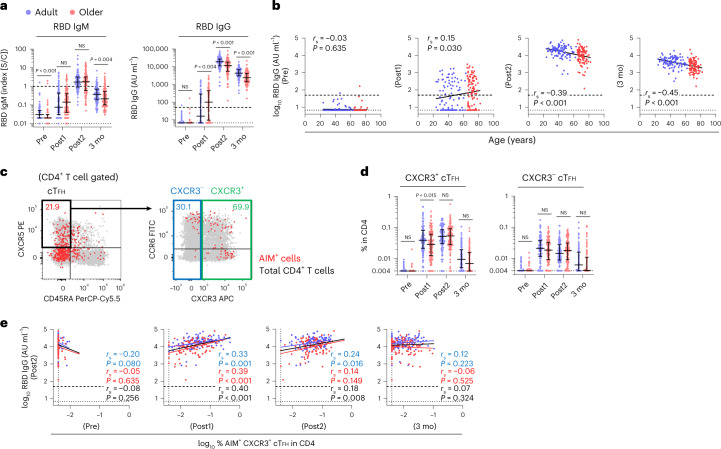
Decreased induction of spike-specific CXCR3^+^ cT_FH_ cells in older adults is associated with their lower IgG titer. **a**, Concentration of anti-RBD IgM and IgG antibodies. **b**, Correlations between the concentration of anti-RBD IgG antibody and age of donors. **c**, Representative flow cytometry plots displaying AIM^+^ CD4^+^ T cells to identify CXCR3^+^ or CXCR3^−^ cT_FH_ (CD45RA^−^CXCR5^+^) subsets. Red and gray dots depict AIM^+^ and total CD4^+^ T cells, respectively, from the same donor after the second dose. Numbers indicate the percentage of AIM^+^ cells in gated fractions. **d**, Frequency of CXCR3^+^ and CXCR3^−^ cT_FH_ cells. **e**, Correlations between the concentration of anti-RBD IgG antibody after the second dose and the percentage of AIM^+^CXCR3^+^ cT_FH_ cells at each time point. In **a** and **d**, the center line and error bars indicate the median and IQR, respectively. In **a**, **b**, **d** and **e**, the dashed and dotted lines indicate cutoff and LOD, respectively. Statistical comparisons across cohorts were performed using the Mann–Whitney test. Spearman’s rank correlation (*r*_s_) was used to identify relationships between two variables, with a straight line drawn by linear regression analysis. For correlation analysis, AIM^+^ and cytokine-positive percentages and concentration of anti-RBD IgG antibody were transformed into logarithmic values. Blue, red and black characters represent the results of statistical test from adults (*n* = 107), older adults (*n* = 109) and both groups (*n* = 216), respectively. Source data

**Extended Data Fig. 9:**
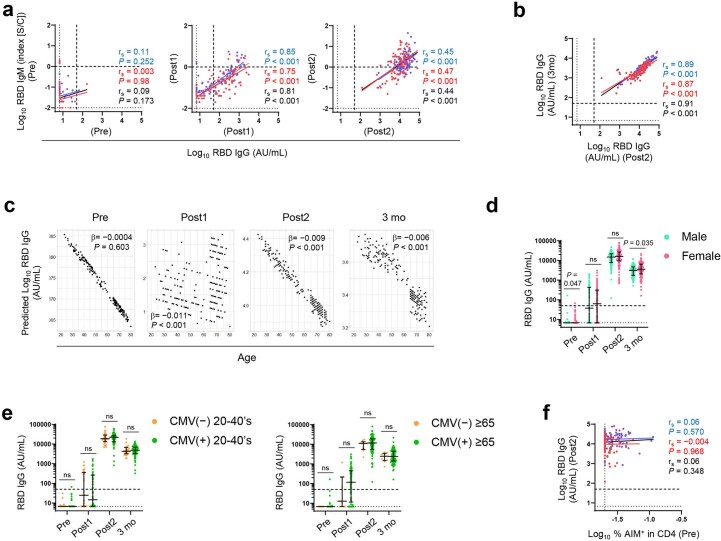
Antibody levels after vaccination. (**a**) Correlation between the concentrations of anti-RBD IgM and anti-RBD IgG antibody at the same sampling points. (**b**) Correlation between the concentrations of anti-RBD IgG antibody after the second dose and at 3 months. (**c**) Predicted concentration of anti-RBD IgG antibody adjusted with days post vaccination was calculated using multiple regression analysis. Regression coefficients (β) of age and *P* values are shown. (**d**, **e**) Concentration of anti-RBD IgG antibody from male (*n* = 99) and female (*n* = 117) donors (**d**) or from CMV-seronegative and -seropositive donors (**e**) in younger adults (20–40 years old) (*n* = 29 and *n* = 54) (e, left) and in older adults (*n* = 9 and *n* = 100) (e, right). (**f**) Correlation between the concentrations of anti-RBD IgG antibody after the second dose and the percentages of AIM^+^ CD4^+^ T cells before vaccination. (**a**, **b**, **d**, **e**, **f**) The dashed and dotted lines indicate cutoff and limit of detection (LOD), respectively. (**a**, **b**, **f**) Concentration of anti-RBD IgM and IgG antibodies were transformed into logarithmic values. Spearman’s rank correlation (r_s_) was used to identify relationships between two variables, with a straight line drawn by linear regression analysis. (**d**, **e**) The centerline and error bars indicate the median and interquartile range (IQR). Statistical comparisons across cohorts were performed using the Mann-Whitney test. ns, not significant. Blue, red, and black dots represent adults (*n* = 107), older adults (*n* = 109), and both groups (*n* = 216), respectively. Source data

Next, we assessed humoral responses. As reported previously^15^, the mRNA vaccination induced robust IgG responses in all donors, and peak levels of anti-receptor-binding domain (RBD) IgG were observed after the second dose in both groups (Fig. 2a). Additionally, IgM concentration peaked after the second vaccine dose, and strong correlations between IgM and IgG responses were observed after the first and second doses (Fig. 2a and Extended Data Fig. 9a), indicating the simultaneous production of IgM and IgG, as reported previously^37^, irrespective of age. Although the antibody levels were highly variable among individuals, even within the same age cohort, we observed a negative correlation between age and peak IgG levels after the second dose (*r*_s_ = −0.39; *P* < 0.001; Fig. 2b). The peak antibody concentrations in the older group (median (IQR), 11,400 (12,645) AU ml^−1^) were approximately 40% lower in median as compared with those in the younger group (median (IQR), 19,000 (16,050) AU ml^−1^; Fig. 2a). Moreover, the median antibody concentration at 3 months decreased to ~20% of those at the peak and were strongly correlated with those after the second dose (Extended Data Fig. 9b), suggesting that the gradual decline in antibody levels mostly reflected a natural decay of the antibodies produced at peak response. Multiple regression analysis confirmed that the predicted IgG adjusted for days since vaccination was negatively associated with age both after the first (*β* = −0.011; *P* < 0.001) and second (*β* = −0.009; *P* < 0.001) doses (Extended Data Fig. 9c). Thus, data showing that the older group had higher IgG levels following the first dose (Fig. 2a) are attributed to the differences in sampling time points. There was a trend of higher IgG levels in females relative to that of males at every time point, with the values being significantly higher at the pre-vaccination stage and at 3 months (Extended Data Fig. 9d), which was consistent with previous findings^38^. No significant differences were observed in IgG titers between age-matched CMV-seropositive and CMV-seronegative individuals (Extended Data Fig. 9e).

To determine a mechanism for the age-related and individual heterogeneities in antibody responses, we investigated associations between antibody levels and CD4^+^ T cell responses. T_FH_ cells are a specified subset of CD4^+^ T cells and play key roles in antibody production and germinal center reactions^39^. We confirmed that the first vaccination rapidly provoked antigen-specific AIM^+^ circulating follicular helper T (cT_FH_) cells (CD3^+^CD4^+^CD45RA^+^CXCR5^+^ T cells) in peripheral blood^40^, while the CXCR3^+^ cT_FH_ subset, which is preferentially induced in the T_H_1 cell condition and associated with efficient antibody responses in the context of infections and vaccinations^32,41,42^, was the major subset of cT_FH_ cells induced by mRNA vaccination (Fig. 2c,d). Notably, the level of CXCR3^+^ cT_FH_ cells, but not CXCR3^−^ cT_FH_ cells, was significantly lower in older adults after the first dose (median (IQR), adults; 0.039% (0.059%) and older adults; 0.029% (0.049%) in total CD4^+^ T cells; *P* < 0.05; Fig. 2d). Furthermore, peak IgG concentration positively correlated with the frequency of CXCR3^+^ cT_FH_ cells after the first dose (*r*_s_ = 0.40; *P* < 0.001 in total donors), but not with that at other time points (Fig. 2e). The frequency of AIM^+^ CD4^+^ T cells before vaccination did not correlate with peak antibody concentration (Extended Data Fig. 9f). These results indicated that older adults show decreased induction of spike-specific CXCR3^+^ cT_FH_ cells in the early stages of vaccine responses, which correlated with their lower IgG responses.

### Lower CD4^+^ T cell induction is associated with fewer adverse events in older adults

**Fig. 3:**
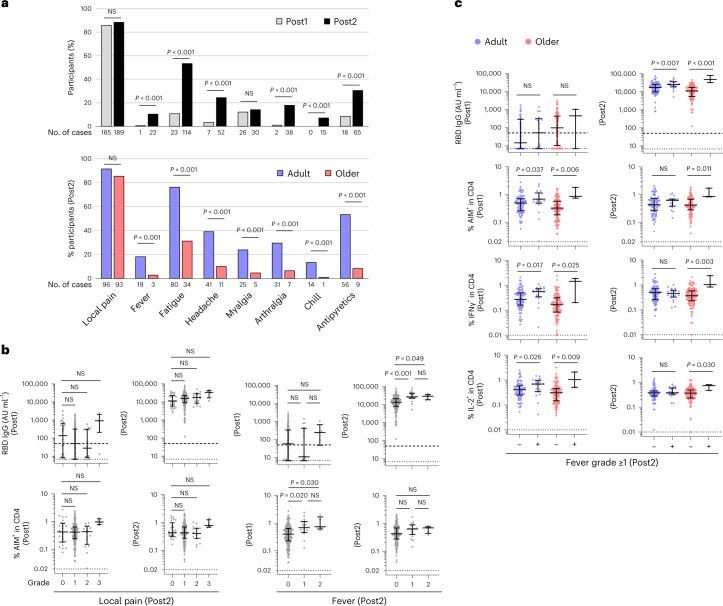
Fewer systemic adverse effects after the second dose are linked to the lower induction of spike-specific CD4^+^ T cells after the first dose. **a**, Frequency of donors with AEs after vaccination. Post1 (*n* = 216) and Post2 (*n* = 214) (upper). Frequency of donors with AEs after the second vaccination in adults and older adults. Adults (*n* = 105) and older adults (*n* = 109) (lower). The number of donors who reported the specified AE is shown below each bar. Fisher’s exact test was used to compare the frequency of participants experiencing AEs by time points and age group. Antipyretics indicate the use of antipyretic medication. **b**, Concentration of anti-RBD IgG antibody and frequency of AIM^+^ CD4^+^ T cells after first and second doses according to the severity of local pain and fever after the second doses in both groups (*n* = 216). Multiple comparisons by grade of adverse event symptoms were performed using the Kruskal–Wallis test with Dunn’s post hoc test. Local pain: grade 0 (*n* = 25), grade 1 (*n* = 169), grade 2 (*n* = 16) and grade 3 (*n* = 4). Fever: grade 0 (*n* = 192), grade 1 (*n* = 17) and grade 2 (*n* = 5). **c**, Concentration of anti-RBD IgG antibody and frequency of AIM^+^ and cytokine^+^ CD4^+^ T cells according to the emergence of fever after the second dose in adults (blue) or older adults (red). A comparison by fever grade in the age group was made using the Mann–Whitney test. Fever grade 0 (fever^−^; *n* = 86) and grade ≥ 1 (fever^+^; *n* = 19) in adults, fever^−^ (*n* = 106) and fever^+^ (*n* = 3) in older adults. In **b** and **c**, the center line and error bars indicate the median and IQR. The dashed and dotted lines indicate cutoff and LOD, respectively. Source data

Although most studies indicate that AEs decrease with age, studies have not addressed whether AEs are associated with T cell responses that can cause systemic effects^43^. In the present study, to minimize individual differences in the definition of AE severity, physicians interviewed each donor and asked about AEs. The grading of AEs is described in the Methods, and the presence of AE (AE^+^) was defined as grade ≥ 1. Local AE (pain at an injection site) was observed in most (>80%) donors in both groups after the first and second doses (Fig. 3a). The percentages of donors positive for systemic AEs (for example, fever, fatigue, headache, arthralgia and chill) were significantly higher after the second than first dose (Fig. 3a), consistent with previous reports^15,20^. Moreover, systemic AEs after the second dose were more commonly observed in adults than in the older group (Fig. 3a). The frequency of participants who self-administered antipyretics after the second dose was higher in adults (53.3%) than in the older group (8.3%; Fig. 3a), suggesting that the systemic AE frequency and degree were substantially underestimated especially in adults.

We then compared the IgG concentration and CD4^+^ T cell response (AIM^+^ cells) among individuals at each grade of local or systemic AE after the second dose. Fever (the most qualitative AE) was selected as the representative systemic AE for the analysis. IgG level and AIM^+^ CD4^+^ T cell frequency were not different among each grade of local pain at both time points (Fig. 3b). By contrast, the fever-positive group (fever ≥ 38 °C) showed significantly higher IgG concentrations following the second dose, as compared with the fever-negative group (Fig. 3b). Furthermore, the fever-positive group showed higher AIM^+^ CD4^+^ T cell frequencies after the first, but not second, dose. No difference was observed in these parameters between grades 1 and 2 (Fig. 3b), while these tendencies were observed in both adults and older groups (Fig. 3c and Supplementary Table 2).

IL-2 and IFN-γ are the major cytokines secreted by CD4^+^ T cells following mRNA vaccination (Fig. 1b)^20,30^. Considering that these cytokines could subsequently induce flu-like symptoms, such as fever or fatigue, upon systemic administration in humans^44^, cytokines rapidly produced upon the second vaccination presumably from the memory CD4^+^ T cells generated after the first dose may be associated with the occurrence of systemic AEs after the second dose. Notably, the percentages of IL-2^+^ and IFN-γ^+^ CD4^+^ T cells were significantly higher in the fever-positive donors after the first dose, rather than after the second dose, in both groups (Fig. 3c). Thus, fever-positive donors have higher induction of spike-specific CD4^+^ T cell responses after the first dose and antibody levels after the second dose. These results suggest that the impaired CD4^+^ T cell induction after the first dose is associated with lower antibody levels and systemic reactogenicity after the second dose, both of which are more frequently observed in older adults.

### Higher PD-1 expression in T_H_1 cells is associated with a lower CD8^+^ T cell response

**Fig. 4:**
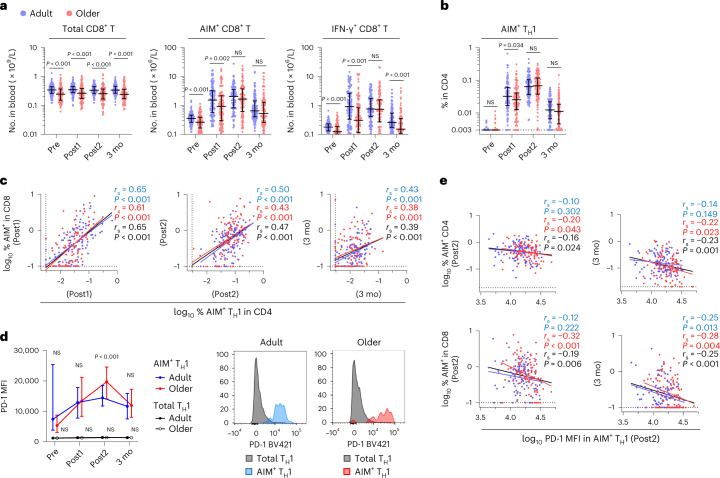
Higher PD-1 expression in spike-specific T_H_1 cells from older adults is correlated with their lower CD8^+^ T cell responses. **a**, Absolute number of total, AIM^+^ (CD69^+^CD137^+^) and IFN-γ^+^ CD8^+^ T cells in the blood. **b**, Frequency of AIM^+^ T_H_1 cells. **c**, Correlations between the percentage of AIM^+^ T_H_1 cells and AIM^+^ CD8^+^ T cells at each time point. **d**, MFI of PD-1 in total or AIM^+^ T_H_1 cells from adults and older adults (left). Representative histogram displaying PD-1 expression in total or AIM^+^ T_H_1 cells after the second dose (right). **e**, Correlations between MFI of PD-1 in AIM^+^ T_H_1 cells after the second dose and the percentages of AIM^+^ CD4^+^ or CD8^+^ T cells after the second dose and 3 months. In **a**, **b** and **d**, the center line and error bars indicate the median and IQR. The dotted line indicates LOD. Statistical comparisons across cohorts were performed using the Mann–Whitney test. Spearman’s rank correlation (*r*_s_) was used to identify relationships between two variables, with a straight line drawn by linear regression analysis. For correlation analysis, percentages of AIM^+^ and cytokine^+^ T cells, and PD-1 MFI were transformed into logarithmic values. Blue, red and black characters represent the results of statistical tests from adults (*n* = 107), older adults (*n* = 109) and both groups (*n* = 216), respectively. Source data

**Extended Data Fig. 10:**
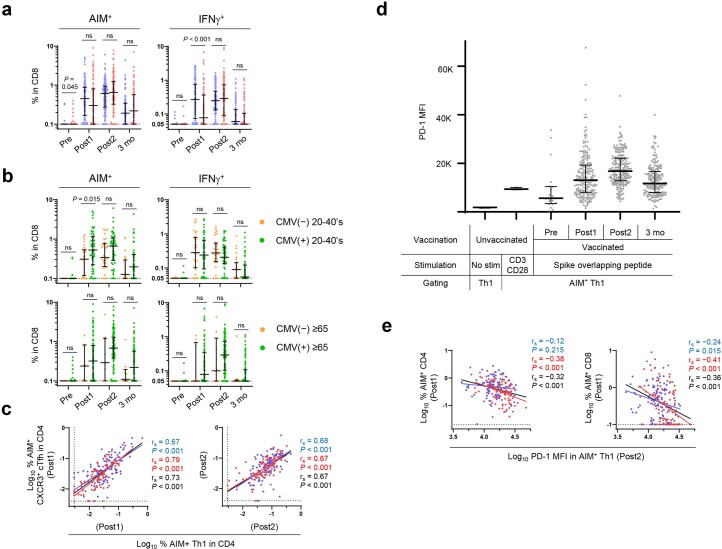
PD-1 expression in spike-specific T_H_1cells and CD8^+^ T cell responses. (**a**) Frequency of AIM^+^ and IFNγ^+^ CD8^+^ T cells. (**b**) Frequency of AIM^+^ and IFNγ^+^ CD8^+^ T cells from CMV-seronegative and -seropositive donors aged 20–40 years (*n* = 29 and *n* = 54, respectively) (upper,) and in older adults (*n* = 9 and *n* = 100, respectively) (lower). (**c**) Correlations between the percentage of AIM^+^ T_H_1(Th1) cells and AIM^+^ CXCR3^+^ cTfh cells after the first and second doses. (**d**) PD-1 MFI in T_H_1(Th1) cells from unvaccinated (*n* = 3) or vaccinated (*n* = 216) donors with or without stimulation using anti-CD3 and anti-CD28 antibodies or spike protein overlapping peptides in AIM assay. (**e**) Correlation between PD-1 MFI in AIM^+^ T_H_1(Th1) cells after the second dose and the percentage of AIM^+^ CD4^+^ and CD8^+^ T cells after the first dose. The dotted line indicates limit of detection (LOD). (**a**, **b**, and **d**) The centerline and error bars indicate the median and IQR. Statistical comparisons across cohorts were performed using the Mann-Whitney test. ns, not significant. (**c**, **e**) Spearman’s rank correlation (r_s_) was used to identify relationships between two variables with a straight line drawn using linear regression analysis. For correlation analysis, percentages of AIM^+^ cells and PD-1 MFI were transformed into logarithmic values. Blue, red, and black dots represent adults (*n* = 107), older adults (*n* = 109), and both groups (*n* = 216), respectively. Source data

Similar to CD4^+^ T cells, frequencies of spike-specific CD8^+^ T cells (AIM^+^ and IFN-γ^+^ CD8^+^ T cells) after the first dose and 3 months were lower in the older group (Extended Data Fig. 10a). This tendency was significant when percentages were calculated in absolute numbers (Fig. 4a), likely due to the significant decrease in CD8^+^ T cell number in peripheral blood with age (Fig. 4a)^33^. No significant differences were found in the frequencies of AIM^+^ and IFN-γ^+^ CD8^+^ T cells between CMV IgG-seropositive and IgG-seronegative individuals in the younger (20–40 years) or the older (≥65 years) group, except for a higher level of AIM^+^ CD8^+^ T cells after the first dose in younger CMV^+^ donors (Extended Data Fig. 10b). Thus, the lower induction and early contraction in older adults upon two doses of mRNA vaccination were also observed in CD8^+^ T cell responses.

T_H_1 cells represent a major helper T cell subset induced by the mRNA vaccine^21,30^, enhancing CD8^+^ T cell expansion and function, and favorable for antiviral immunity. We confirmed that AIM^+^ T_H_1 cells (CD45RA^−^CXCR5^−^CXCR3^+^CCR6^−^CD4^+^ T cells) increased significantly after vaccination; however, the first vaccination elicited fewer spike-specific T_H_1 cells in older adults than in adults (Fig. 4b), consistent with the lower IL-2^+^ and IFN-γ^+^ CD4^+^ T cells in older adults (Fig. 1b). The frequencies of AIM^+^ T_H_1 cells were well correlated with those of AIM^+^ CD8^+^ T cells at all time points in both groups (Fig. 4c), confirming the association of T_H_1 and CD8^+^ T cell responses^21,28^. The frequencies of spike-specific T_H_1 cells were also correlated with those of CXCR3^+^ cT_FH_ cells (Extended Data Fig. 10c), suggesting that donors with low T_H_1 cells may also have low CXCR3^+^ cT_FH_ cells.

To gain an insight into a possible mechanism underlying the low CD4^+^ T cell responses and subsequent compromised antigen-specific CD8^+^ T cell responses in the older group, we focused on PD-1, which is upregulated after T cell activation and negatively regulates immune responses to prevent excess and/or autoimmune reactions^45^. PD-1 expression (assessed via MFI) in total T_H_1 cells was unchanged in both groups during the observation period (Fig. 4d), while that in spike-specific AIM^+^ T_H_1 cells gradually increased, peaked after the second dose, and decreased at 3 months (Fig. 4d). PD-1 expression in AIM^+^ T_H_1 cells from pre-vaccinated, peptide-stimulated samples was slightly higher than that in total T_H_1 cells from unvaccinated samples, suggesting that the peptide stimulation during the AIM assay slightly upregulated PD-1 expression (Fig. 4d). However, the expression levels of PD-1 in AIM^+^ T_H_1 cells from vaccinated PBMCs were much higher than those from unvaccinated, even with the strong T cell receptor (TCR) stimulation by anti-CD3 and anti-CD28 antibodies (Extended Data Fig. 10d), strongly suggesting that the PD-1 levels detected in the AIM assay reflect the gradual upregulation and downregulation of PD-1 expression in vivo. Importantly, the PD-1 expression in spike-specific T_H_1 cells was significantly higher in older adults after the second dose than in adults (Fig. 4d), while the PD-1 expression levels at peak response were negatively correlated with the frequencies of spike-specific CD4^+^ and CD8^+^ T cells in older adults (Fig. 4e and Extended Data Fig. 10e). These results suggest that T_H_1 cells in older adults tended to express PD-1 at higher levels via continuous antigen stimulation upon two doses of mRNA vaccination, while PD-1 expression levels at peak responses were associated with lower CD4^+^ and CD8^+^ T cell responses in older adults.

## Discussion

In this study, we investigated the detailed trajectory of CD4^+^ T cell responses to mRNA vaccines in older individuals and their association with humoral and cellular immunity as well as reactogenicity. We found that the older population showed a significantly lower induction of T_H_1 cells and CXCR3^+^ cT_FH_ cells, after the first dose, rather than the second dose, which correlated with the lower CD8^+^ T cell and peak antibody responses, respectively. Because the frequencies among antigen-specific CXCR3^+^ cT_FH_, T_H_1 and CD8^+^ T cells showed a positive correlation even in older adults, the lower induction of one T cell subset was not attributed to the biased T cell responses to other directions or the uncoordinated responses as previously reported in older patients with COVID-19 disease^32^. Rather, our results strongly suggest that older adults are more likely to exhibit lower elicitation of T_H_1-skewed CD4^+^ T cell responses that coordinate immune responses^20,21^, thereby attenuating all arms of adaptive immunity. Our results may be consistent with a previous study indicating that the rapid induction of antigen-specific CD4^+^ T cells was associated with coordinated humoral and cellular immunity^21^. Importantly, a poor response to the first dose was also observed in patients harboring solid cancer with lower antibody responses^46,47^. These results strongly suggested that CD4^+^ T cell responses to the first dose is key to the improved consequences of vaccination, and that older individuals tend to have a defect in this process. Moreover, even in the case of SARS-CoV-2 infection, a delay in the development of T_FH_ cells and subsequent neutralizing antibodies correlates with fatal COVID-19 disease^48^. Thus, the delayed induction of CD4^+^ T cell responses to either vaccination or even viral infection could be a predictive factor for compromised immune responses.

The mechanisms underlying lower CD4^+^ T cell responses after the first dose in older adults remain to be determined. A previous report suggested a correlation between the frequencies of preexisting SARS-CoV-2 spike-reactive memory CD4^+^ T cells and vaccine-induced CD4^+^ responses upon a low dose of vaccine^30^. We also observed a correlation of spike-specific CD4^+^ T cell frequencies between the pre-vaccinated stage and after the first dose in older adults. However, this correlation was weak (*r*_s_ = 0.23), while the frequency of preexisting CD4^+^ T cells did not correlate with those of spike-specific CD4^+^ T cells or IgG titer after the second dose, suggesting little impact of cross-reactive T cells on the efficient induction of CD4^+^ T cells and humoral responses, at least with the current standard two doses of vaccines. Rather, we observed that the cell sizes of spike-specific CD4^+^ T cells after the first dose in the older group were significantly smaller than those in adults, suggesting an inefficient activation of CD4^+^ T cells. Although the CD4^+^ T cell compartment is relatively well maintained qualitatively and quantitatively compared with CD8^+^ T cells, especially in humans^12,33^, several T cell intrinsic defects, such as T cell receptor desensitization or epigenetic changes, have been reported^11,49^. T cell-extrinsic factors, including a defect in antigen-presenting cells with age, were also observed^50^. Further investigation is warranted to determine which factor primarily contributes to the lower induction of CD4^+^ T cell responses to mRNA vaccines.

Older adults also showed the early contraction of spike-specific T cells, suggesting an accelerated cell death^51^. However, the vaccine-induced CD4^+^ T cells were phenotypically similar in the two groups and mainly and stably mapped to CM subsets during the study period. Notably, PD-1 expression in spike-specific T_H_1 cells at a peak response was higher in older adults. Although it is currently unclear whether the higher PD-1 expression levels reflect higher activation or exhaustion, the PD-1 expression in older adults was associated with less spike-specific CD4^+^ and CD8^+^ T cell expansion and maintenance, suggesting an inhibitory role of PD-1. On the other hand, considering that the PD-1 expression at a peak response was also negatively correlated with CD4^+^ and CD8^+^ T cell frequencies after the first dose (Extended Data Fig. 10e), the higher expression of PD-1 at peak response and the lower induction and early contraction of T cell responses could be causally unrelated, yet both are the characteristics of vaccine-induced T cell responses in older adults.

In addition to the age-associated differences, we observed considerable individual variability, which showed a >10-fold difference in the frequencies of spike-specific T cells and antibody levels, even within the same age cohort. Most studies have found that CMV infection accelerates immune senescence and has a negative effect on vaccine outcomes^52,53^, whereas CMV infection has a positive effect on the vaccine response, especially in young individuals^54^, possibly due to the basal activation of the innate immunity. However, we observed no obvious differences in antibody levels or T cell responses between CMV-seropositive and CMV-seronegative individuals of both younger and older cohorts. Considering the long-life expectancy of Japanese individuals, and that our cohort only included healthy individuals, the possible impacts of CMV infection may be difficult to observe in this study. It is also possible that two doses of mRNA vaccination generate such robust immune responses that the negative impact is not detectable. Further studies to elucidate whether the low responders in younger adults show accelerated T cell and/or immune aging and how to predict the outcome of immune responses and define the immune age will be critical to understanding the mechanisms associated with the large individual variations in immune responses to the mRNA vaccine.

Another key observation was the association between systemic reactogenicity and T cell responses, which suggests that the high number of effector and memory T cells efficiently induced by the first dose, may rapidly produce large amounts of cytokines in response to the second dose. IFN-γ^+^ cells were most significantly reduced in CD4^+^ T cells from the older group after the first dose. IFN-γ is a potent inducer of flu-like symptoms^44^ and also plays an important role in the coordination of immune responses^55^, which likely explains the correlations observed among T cell responses after the first dose, AEs after the second dose and peak IgG levels. This hypothesis is consistent with a previous study indicating that IFN-γ, which is mainly produced by T_H_1 cells, and not type I interferons, is the first and primary cytokine demonstrating marked increases at day 1 after the second dose, suggestive of a systemic effect of IFN-γ^56^. The fever-negative group included individuals with high levels of T cell responses and antibody titers, which were much higher than the median of the fever-positive group. In the present cohort, ~30% of participants self-administered antipyretics after the second dose, which might have at least partially affected the results. Altogether, these data strongly suggest that a high degree of systemic reactogenicity following delivery of the mRNA vaccine might be an indicator of a strong CD4^+^ T cell response, and this leads to efficient and coordinated vaccine-induced immune responses, which was less frequently observed in older adults.

This study has several limitations. First, although individuals ≥ 65 years of age are commonly defined as older adults, there is no clear medical or biological evidence to support this definition. Second, we did not analyze antigen-presenting cells and B cells that are also critical for vaccine-induced immunity. Third, we only investigated spike-specific T cells in peripheral blood; therefore, it remains unclear whether T cells in secondary lymphoid organs, where actual immune responses occur, differ between the two groups. Moreover, although the AIM assay is widely used for detecting antigen-specific T cells, short-term TCR stimulation during the assay could affect the T cell phenotypes. Fourth, we evaluated only anti-RBD antibody titer but not neutralizing activity, in which cT_FH_ cells play an important role^39^, although these two parameters are highly correlated^30,57^. Finally, we provided evidence only of associations between CD4^+^ T cell responses with antibody and CD8^+^ T cell responses as well as AEs. Further studies are warranted to investigate causal relationships among these parameters.

In conclusion, we demonstrated the characteristics of immune responses to the two doses of mRNA vaccine BNT162b2 in older individuals (aged ≥ 65 years), revealing a lower induction and early contraction of antigen-specific T cells. Specifically, the decreased induction of CD4^+^ T cells after the first dose may provide a useful (although incomplete) proxy for the lower antibody response, CD8^+^ T cell response and systemic reactogenicity. This study provides insights into the development of vaccines with higher efficacy and the establishment of a vaccine schedule suitable for the older population.

## Methods

### Study design

This study was reviewed and approved by the Kyoto University Graduate School and Faculty of Medicine, Ethics Committee (R0418). Two hundred and twenty-five participants applied to participate in the study. At the time of enrollment, all donors provided written informed consent, in accordance with the Declaration of Helsinki. Donors were required to be aged ≥20 years. For the first and second doses, only participants who received Pfizer BNT162b2 were considered eligible. Participants received a BNT162b2 prime dose on day 0 and a boost dose on around day 21, as recommended by the manufacturer. Blood sampling points were set with an allowance (Extended Data Fig. 1a). Participants were followed up for medical inquiries including AEs at each visit. Grading of AEs was performed according to the United States Food and Drug Administration recommendations and previous studies related to BNT162b2 (refs. 15, 20); specifically, pain at the injection site was graded as grade 1 (does not interfere with activity), grade 2 (interferes with activity), grade 3 (prevents daily activity) or grade 4 (led to an emergency department visit or hospitalization), and fever was graded as grade 1 (38.0–38.4 °C), grade 2 (38.5–38.9 °C), grade 3 (39.0–40.0 °C) or grade 4 (>40.0 °C).

All donors were otherwise healthy and did not report any ongoing severe medical conditions, including cancer, gastrointestinal, liver, kidney, cardiovascular, hematologic or endocrine diseases. Those taking medications that may affect the immune system, including steroids or immunomodulatory drugs, were excluded. Blood samples were collected at the Ki-CONNECT and Clinical BioResource Center (CBRC) at Kyoto University Hospital. Samples were de-identified using an anonymous code assigned to each sample. Only samples without bloodborne pathogens, including HIV, HTLV-1, HBV and HCV, were used for subsequent experiments. Six participants did not meet the eligibility criteria, and a total of 219 individuals consisting of 107 adults (aged less than 65 years, workers in Kyoto University Hospital) and 112 older individuals (aged more than 65 years, general population) were enrolled in the study. According to the study protocol, only older participants were compensated for the cost of public transportation. As healthcare workers are generally careful about their health but tend to be exposed to various pathogens while they work, their immune response may not reflect that of the general population. As older adults who participated in this study were recruited openly using the internet, they might have made a more active effort to safeguard their health, and may have been more likely to report adverse events than the general older adult population. Two patients were lost to follow-up, and one was stopped because of mRNA-1273 injection as the primary and boost vaccination (Extended Data Fig. 1b). Their characteristics, including age, sex and serology, are summarized in Table 1.

### Peripheral blood mononuclear cells isolation, cryopreservation and thawing

Whole blood was drawn into Vacutainer CPT Cell Preparation Tubes with sodium citrate (BD Biosciences), according to the manufacturer’s instructions, and processed within 2 h to isolate PBMCs. Isolated PBMCs were resuspended in CELLBANKER 1 (ZENOGEN PHARMA) at a concentration of 8 × 10^6^ cells per milliliter and aliquoted in 250 or 500 μl per cryotube. Samples were stored at −80 °C on the day of collection and in liquid nitrogen until used for the assays. Cryopreserved PBMCs were thawed in pre-warmed X-VIVO15 (LONZA) without serum. After centrifugation, cells were washed once and used directly for assays.

### Complete blood counts

Whole blood was collected in ethylenediaminetetraacetic-2Na tubes. The analysis was performed using an Automated Hematology Analyzer XN-9000 (Sysmex) at the Department of Clinical Laboratory, Kyoto University Hospital.

### Serology

Whole blood was collected in a Venoject VP-P075K (Terumo) blood collection vessel for serum isolation. The serum separator tubes were centrifuged for 4 min at 1,100*g* at 4 °C. The serum was then removed from the upper portion of the tube, aliquoted, and stored at −80 °C.

Anti-SARS-CoV-2 (N protein) IgM/IgG levels in the serum were measured using an Elecsys Anti-SARS-CoV-2 with cobas 8000 (Roche Diagnostics KK) at the Department of Clinical Laboratory, Kyoto University Hospital. Anti-SARS-CoV-2 RBD IgM and IgG levels were measured at LSI Medience (Tokyo, Japan) using ARCHITECT SARS-CoV-2 IgM and ARCHITECT SARS-CoV-2 IgG II Quant (Abbott), respectively. Anti-CMV IgG levels were measured using a chemiluminescence immunoassay (CLIA) at LSI Medience (Tokyo, Japan). The cutoff values for anti-SARS-CoV-2 (N protein) IgM/IgG, anti-SARS-CoV-2 RBD IgM, IgG and Anti-CMV IgG were 1.0 cutoff index (COI), 1.0 (COI), 50 (AU ml^−1^) and 6.0 (AU ml^−1^), respectively.

### Peptide pools

PepTivator SARS-CoV-2 Prot_S Complete peptide pools (Miltenyi Biotech) were diluted in distilled water (DW) and used for spike-specific T cell stimulation. The S peptide pool contains 15-mer peptides that overlap by 11 amino acids and cover the complete protein-coding sequence (amino acids 5–1,273) of the surface or spike glycoprotein (S) of SARS coronavirus 2 (GenBank MN908947.3, Protein QHD43416.1). Peptide pools were added to the culture medium at a final concentration of 0.6 nmol ml^−1^.

### Activation-induced marker assay

PBMCs were cultured in 100 µl of X-VIVO15 medium supplemented with 5% human AB serum for 23 h at 37 °C in the presence of SARS-CoV-2 peptide pools (0.6 nmol ml^−1^) and CD40 blocking antibody (0.5 µg ml^−1^, Miltenyi Biotech) in 96-well U-bottom plates (Corning) at 1 × 10^6^ PBMCs per well. An equal volume of DW was used as a negative control. For T cell receptor stimulation, 4 × 10^5^ PBMCs were cultured in a 96-well flat-bottom plate (Corning) in the presence of coated anti-CD3 (0.5 μg per well; clone UCHT1, BioLegend) and soluble anti-CD28 antibodies (2 μg ml^−1^; clone CD28.2, BioLegend). After stimulation, cells were stained with fluorochrome-conjugated surface antibodies at pre-titrated concentrations in the presence of FcR blocking (Miltenyi Biotech) for 20 min at 4 °C. The cells were then washed and stained with Ghost Dye Red 710 (TONBO) to discriminate between viable and non-viable cells. After the final wash, the cells were resuspended in 100 µl PBS with 2% FBS (FACS buffer) for flow cytometry. The antibodies used in the AIM assay are listed in Supplementary Table 1. Spike-specific AIM^+^ T cells were defined based on the coexpression of CD154 (CD40L) and CD137 for CD4^+^ T cells and CD137 and CD69 for CD8^+^ T cells (Extended Data Fig. 2b). Antigen-specific responses were quantified as the frequency of AIM^+^ cells in stimulated samples, with background subtraction from paired DW controls (Extended Data Fig. 2b). The LOD for AIM^+^ cells was calculated as previously described^58^. Values ≥ LOD (AIM^+^ CD4^+^ T cells; 0.02% and AIM^+^ T_H_1 cells; 0.003%) and stimulation index (SI) > 2 were considered for the phenotypic analysis of antigen-specific T cells, such as Boolean analysis, FSC-A MFI, PD-1 MFI, and T cell subset identification. The SI was calculated by dividing the percentage of AIM^+^ cells after SARS-CoV-2 peptide pool stimulation with that after DW treatment. If the percentage of AIM^+^ cells after DW stimulation equaled 0, the minimum value across each cohort was used instead.

### Intracellular cytokine staining assay

Similarly to the AIM assay, PBMCs were cultured in 100 µl of X-VIVO15 medium supplemented with 5% human AB serum for 23 h at 37 °C in the presence of SARS-CoV-2 peptide pools (0.6 nmol ml^−1^) at 1 × 10^6^ PBMCs per well. An equal volume of DW was used as the negative control. Four hours before staining and fixation, brefeldin A (BioLegend; 1:1,000 dilution) was added to the medium. After stimulation, cells were stained with fluorochrome-conjugated surface antibodies at pre-titrated concentrations in the presence of FcR blocking for 20 min at 4 °C. Cells were then washed and stained with Ghost Dye Red 710 to discriminate between viable and non-viable cells. The stained cells were then fixed with IC fixation buffer (Thermo Fisher Scientific) for 30 min at 4 °C and washed twice with permeabilization buffer (Thermo Fisher Scientific) and subsequently stained for intracellular IL-2, IL-4, IL-17A, IFN-γ, TNF, perforin and granzyme (1:100 dilution each) for 30 min at room temperature. After the final wash, the cells were resuspended in 100 µl of FACS buffer for flow cytometry. The antibodies used in ICS assays are listed in Supplementary Table 1. Vaccine-induced cytokine-producing T cells were quantified as the frequency of cytokine-positive cells in stimulated samples, with background subtraction from paired DW controls (Extended Data Fig. 3b). LOD for each cytokine^+^ cells was calculated as previously described^58^.

### Flow cytometry and Flow Cytometry Standard data analysis

All AIM and ICS assay samples were acquired using Northern Light 3000 and SpectroFlo software version 2.2 (Cytek Biosciences). Flow Cytometry Standard (FCS) 3.0 data files were exported and analyzed using FlowJo software version 10.8.1. The detailed gating strategies for individual markers are described in Extended Data Figs. 2 and 3. The subset definitions and gating strategies are outlined in the text or figures.

The absolute number of a defined subset of CD4^+^ and CD8^+^ T cells was obtained by multiplying the number of CD4^+^ or CD8^+^ T cells by the percentage of the corresponding subsets. The percentages used to calculate the number of each T cell fraction were obtained from samples of T cells stimulated with DW (negative control), in which the proportional changes in each T cell subset were minimal between before and after 23 h in culture (data not shown). Proportions of multiple cytokine-expressing CD4^+^ T cells (IFN-γ, IL-2, IL-4, IL-17A and TNF-α) were assessed by Boolean analysis as reported previously^29^. LOD for all the background-subtracted subpopulations of cytokine-positive cells was calculated as previously described^58^. Values ≥ LOD and SI > 2 were considered for the analysis. Relative proportions of number of functions are displayed as a pie chart.

All flow cytometry samples were analyzed using eight separate experiments; samples from each donor obtained at all time points (Pre, Post1, Post2 and 3 mo) were simultaneously analyzed, and those from adults and older adults were equally included in one experiment. To define inter-assay variation for AIM and ICS assays, PBMCs from unvaccinated donors (Lot A for exp nos. 1–7 and Lot B for exp nos. 6–8) stimulated with PHA (positive control) or DW (negative control) were included in each independent experiment as an internal quality control.

### opt-SNE and FlowSOM

Dimensionality reduction and cell clustering of multicolor flow cytometry data obtained from AIM assays was performed using opt-SNE and FlowSOM in OMIQ software. FCS 3.0 data from all donors were imported. Up to 40 AIM^+^ CD4^+^ T cells were sub-sampled and merged for analysis. The subsampling counts were derived from the 25 percentiles in the corresponding subset, which could pool AIM^+^ cells evenly from most donors. The markers applied to the opt-SNE and FlowSOM are described in the figures. The parameters used for opt-SNE were: max iterations, 1,000; opt-SNE end, 5,000; perplexity, 30; theta, 0.5; components, 2; random seed, 6,925; verbosity, 25; and for FlowSOM: xdim, 10; ydim, 10; rien, 10; comma-separated *k* values, 20, 25; and random seed, 6,793.

### Statistics and reproducibility

Statistical analyses were performed using GraphPad Prism 9.0. Statistical details, such as groups, statistical tests, and significance values of the results are provided in the respective figure legends. All statistical tests were two-sided, and *P* values < 0.05 were considered statistically significant. According to Guilford’s Rule of Thumb, a correlation coefficient (*r*_s_) of ≥ 0.2 and a *P* value < 0.05 are considered correlated^59^. Nonparametric statistical tests were used because the data were not assumed to be normally distributed. No statistical methods were used to predetermine the sample size, but the sample size in this study is similar to those reported in previous publications^16,60^. As this was an observational study, randomization was not applied to this study. The investigators were not blinded to allocation during this study or during the outcome assessment.

### Reporting summary

Further information on research design is available in the Nature Portfolio Reporting Summary linked to this article.

## Supplementary information

Supplementary Information Supplementary Tables 1 and 2.

Reporting Summary

## Data Availability

Source data are provided with this paper. Any additional raw and supporting data required are available from the corresponding author on request.

## Source data

Source Data Fig. 1 Statistical source data.
Source Data Fig. 2 Statistical source data.
Source Data Fig. 3 Statistical source data.
Source Data Fig. 4 Statistical source data.
Source Data Extended Data Fig. 4 Statistical source data.
Source Data Extended Data Fig. 5 Statistical source data.
Source Data Extended Data Fig. 6 Statistical source data.
Source Data Extended Data Fig. 7 Statistical source data.
Source Data Extended Data Fig. 8 Statistical source data.
Source Data Extended Data Fig. 9 Statistical source data.
Source Data Extended Data Fig. 10 Statistical source data.
