# Author Correction: Impaired CD4^+^ T cell response in older adults is associated with reduced immunogenicity and reactogenicity of mRNA COVID-19 vaccination

**DOI:** 10.1038/s43587-023-00426-w

**Published:** 2023-05-02

**Authors:** Norihide Jo, Yu Hidaka, Osamu Kikuchi, Masaru Fukahori, Takeshi Sawada, Masahiko Aoki, Masaki Yamamoto, Miki Nagao, Satoshi Morita, Takako E. Nakajima, Manabu Muto, Yoko Hamazaki

**Affiliations:** 1 Department of Life Science Frontiers, Center for iPS Cell Research and Application (CiRA), Kyoto University, Kyoto, Japan; 2 Alliance Laboratory for Advanced Medical Research, Graduate school of Medicine, Kyoto University, Kyoto, Japan; 3 Department of Biomedical Statistics and Bioinformatics, Graduate School of Medicine, Kyoto University, Kyoto, Japan; 4 Department of Therapeutic Oncology, Graduate School of Medicine, Kyoto University, Kyoto, Japan; 5 Clinical Bio-Resource Center, Kyoto University Hospital, Kyoto, Japan; 6 Department of Early Clinical Development, Graduate school of Medicine, Kyoto University, Kyoto, Japan; 7 Kyoto Innovation Center for Next Generation Clinical Trials and iPS Cell Therapy (Ki-CONNECT), Kyoto University Hospital, Kyoto, Japan; 8 Department of Clinical Laboratory Medicine, Graduate School of Medicine, Kyoto University, Kyoto, Japan; 9 Laboratory of Immunobiology, Graduate school of Medicine, Kyoto University, Kyoto, Japan

**Keywords:** Lymphocytes, Ageing, Infection

Correction to: *Nature Aging* 10.1038/s43587-022-00343-4. Published online 12 January 2023.

In the version of this article initially published, two transcriptional errors were present in the Results subsection “Lower CXCR3^+^ T_FH_ cell induction is correlated with lower IgG levels in older adults.” Starting from the fifth sentence in the right-hand column on page 84, the values “18,200 (5,845)” and “27,200 (7,850)” have been replaced with “11,400 (12,645)” and “19,000 (16,050),” respectively. In the seventh sentence from the bottom in the left-hand column on page 85, “−0.40” has been corrected to “0.40” as the *r*_s_ value. The original data published in the corresponding figures (Fig. 2a,e) and source data file were correct and the conclusions of the paper remain unaffected. The errors have been corrected in the HTML and PDF versions of the article.

