# Supplementary material for: Impaired CD4+ T cell response in older adults is associated with reduced immunogenicity and reactogenicity of mRNA COVID-19 vaccination
Source: Nat Aging. 2023 Jan 12;3(1):82–92. doi: 10.1038/s43587-022-00343-4 (PMC10154196; doi:10.1038/s43587-022-00343-4)
Supplement: Supplementary file 1 — Supplementary Tables 1 and 2. [file 43587_2022_343_MOESM1_ESM.pdf]

nature aging

Article https://doi.org/10.1038/s43587-022-00343-4

Impaired CD4$^+$ T cell response in older adults is associated with reduced immunogenicity and reactogenicity of mRNA COVID-19 vaccination

In the format provided by the authors and unedited

Table S1. Antibody list used for AIM and ICS assay.

AIM

Marker	Fluorochrome	Clone	Manufacturer	Dilution
PD-1	BV421	EH12.2H7	Biolegend	1:100
CD57	BV510	QA17A04	Biolegend	1:2500
CD8	BV570	RPA-T8	Biolegend	1:500
CD154	BV605	24-31	Biolegend	1:100
CD69	BV650	FN50	Biolegend	1:100
CD28	BV750	CD28.2	Biolegend	1:50
CD95	BV785	DX2	Biolegend	1:200
CCR6	FITC	G034E3	Biolegend	1:100
CD4	AF532	RPA-T4	Invitrogen	1:100
CXCR5	PE	J252D4	Biolegend	1:100
CD45RA	PerCP-Cy5.5	HI100	Biolegend	1:100
CD3	PerCP-eF710	OKT3	Invitrogen	1:250
CD137	PE-Cy7	4B4-1	Biolegend	1:100
CXCR3	APC	G025H7	Biolegend	1:50
GD710	AF700	-	TONBO	1:1000
CCR7	APC-Cy7	G043H7	Biolegend	1:100

ICS

Marker	Fluorochrome	Clone	Manufacturer	Dilution
Perforin	BV421	dG9	Biolegend	1:100
CD57	BV510	QA17A04	Biolegend	1:2500
CD8	BV570	RPA-T8	Biolegend	1:500
CD45RA	BV605	HI100	Biolegend	1:100
TNFa	BV650	MAb11	Biolegend	1:100
CD28	BV750	CD28.2	Biolegend	1:50
CD95	BV785	DX2	Biolegend	1:200
CCR7	FITC	G043H7	Biolegend	1:100
CD4	AF532	RPA-T4	Invitrogen	1:100
IL-4	PE	MP4-25D2	Biolegend	1:100
Granzyme	PerCP-Cy5.5	QA18A28	Biolegend	1:100
CD3	PerCP-eF710	OKT3	Invitrogen	1:250
IL-2	PE-Cy7	MQ1-17H12	Biolegend	1:100
IFNɣ	APC	4S.B3	Biolegend	1:100
GD710	AF700	-	TONBO	1:1000
IL-17A	APC-Cy7	BL168	Biolegend	1:100

Table S2. Antibody levels and CD4$^+$ T-cell responses of individuals by subgrouping them according to the grade of fever after the 2nd dose.

Post 1

Parameters (Post1)	Grade of fever (Post2)	Adults n = 107 Median (IQR)	Older adults n = 109 Median (IQR)
RBD IgG	0	14.1 (280)	96.3 (419.2)
	1	10.95 (462)	225.4 (437.2)
	2	166.8 (252.5)	1030
AIM$^+$ CD4 (%)	0	0.49 (0.45)	0.33 (0.39)
	1	0.60 (0.71)	1.33 (0.93)
	2	1.00 (1.26)	0.74
IFNγ$^+$ CD4 (%)	0	0.28 (0.34)	0.17 (0.24)
	1	0.58 (0.44)	1.70 (0.40)
	2	0.44 (0.38)	0.2
IL-2$^+$ CD4 (%)	0	0.41 (0.33)	0.31 (0.31)
	1	0.69 (0.71)	1.6 (1.10)
	2	0.60 (0.93)	0.51

Post 2

Parameters (Post2)	Grade of fever (Post2)	Adults n = 107 Median (IQR)	Older adults n = 109 Median (IQR)
RBD IgG	0	17550 (15862)	11200 (12195)
	1	25100 (14000)	64000 (32000)
	2	23800 (19500)	32200
AIM$^+$ CD4 (%)	0	0.43 (0.45)	0.42 (0.4)
	1	0.63 (0.32)	1.29 (0.86)
	2	0.59 (0.28)	0.83
IFNγ$^+$ CD4 (%)	0	0.43 (0.35)	0.37 (0.36)
	1	0.54 (0.34)	1.6 (1.57)
	2	0.35 (0.17)	1
IL-2$^+$ CD4 (%)	0	0.37 (0.23)	0.38 (0.26)
	1	0.33 (0.12)	0.58 (0.2)
	2	0.34 (0.41)	0.33

Number of donors:

Adults (< 65 years) : grade 0 (n = 86), grade 1 (n = 15), grade 2 (n = 4)

Older adults (≥ 65 years) : grade 0 (n = 106), grade 1 (n = 2), grade 2 (n = 1)
