# Supplementary material for: Impaired CD4+ T cell response in older adults is associated with reduced immunogenicity and reactogenicity of mRNA COVID-19 vaccination
Source: Nat Aging. 2023 Jan 12;3(1):82–92. doi: 10.1038/s43587-022-00343-4 (PMC10154196; doi:10.1038/s43587-022-00343-4)
Supplement: Supplementary file 2 — Reporting Summary [file 43587_2022_343_MOESM2_ESM.pdf]

nature portfolio

Corresponding author(s): Yoko Hamazaki

Last updated by author(s): Nov 7, 2022

Reporting Summary

Nature Portfolio wishes to improve the reproducibility of the work that we publish. This form provides structure for consistency and transparency in reporting. For further information on Nature Portfolio policies, see our Editorial Policies and the Editorial Policy Checklist.

Statistics

For all statistical analyses, confirm that the following items are present in the figure legend, table legend, main text, or Methods section.

n/a	Confirmed	
☐	☒	The exact sample size (*n*) for each experimental group/condition, given as a discrete number and unit of measurement
☐	☒	A statement on whether measurements were taken from distinct samples or whether the same sample was measured repeatedly
☐	☒	The statistical test(s) used AND whether they are one- or two-sided<br>*Only common tests should be described solely by name; describe more complex techniques in the Methods section.*
☐	☒	A description of all covariates tested
☐	☒	A description of any assumptions or corrections, such as tests of normality and adjustment for multiple comparisons
☐	☒	A full description of the statistical parameters including central tendency (e.g. means) or other basic estimates (e.g. regression coefficient) AND variation (e.g. standard deviation) or associated estimates of uncertainty (e.g. confidence intervals)
☐	☒	For null hypothesis testing, the test statistic (e.g. *F*, *t*, *r*) with confidence intervals, effect sizes, degrees of freedom and *P* value noted<br>*Give P values as exact values whenever suitable.*
☒	☐	For Bayesian analysis, information on the choice of priors and Markov chain Monte Carlo settings
☒	☐	For hierarchical and complex designs, identification of the appropriate level for tests and full reporting of outcomes
☐	☒	Estimates of effect sizes (e.g. Cohen's *d*, Pearson's *r*), indicating how they were calculated

Our web collection on statistics for biologists contains articles on many of the points above.

Software and code

Policy information about availability of computer code

Data collection	Flow cytometry data were acquired using NL-3000 and SpectroFlo software v2.2 (Cytek). This paper does not report original code.
Data analysis	FCS 3.0 data files were exported and analyzed using FlowJo software version 10.8.1. Microsoft Excel version 2210 was used to organize data and donor information. GraphPad Prism 9.0 was used to analyze data and create figures. opt-SNE and FlowSOM analysis were performed using OMIQ software.

For manuscripts utilizing custom algorithms or software that are central to the research but not yet described in published literature, software must be made available to editors and reviewers. We strongly encourage code deposition in a community repository (e.g. GitHub). See the Nature Portfolio guidelines for submitting code & software for further information.

Data

Policy information about availability of data

All manuscripts must include a data availability statement. This statement should provide the following information, where applicable:

- Accession codes, unique identifiers, or web links for publicly available datasets
- A description of any restrictions on data availability
- For clinical datasets or third party data, please ensure that the statement adheres to our policy

All data reported in this article are provided as Source Data. Any additional raw and supporting data are available from the corresponding author upon request.

Human research participants

Policy information about studies involving human research participants and Sex and Gender in Research.

Reporting on sex and gender	The biological attribute of sex was used and this study included male (n = 99) and female (n = 117), based on self-reporting. Analysis comparing values between the sexes are presented in Extended Data Fig. 5a and Extended Data Fig. 9d. Raw data with disaggregated sex are presented in Source Data of Extended Data Fig. 5 and Fig. 9.
Population characteristics	Donors' characteristics, including age, sex, and serology, are summarized in Table 1.
Recruitment	Two hundred and twenty-five participants applied to participate in the study. At the time of enrollment, all donors provided written informed consent, in accordance with the Declaration of Helsinki. Donors were required to be ≥ 20 year. For the first and second doses, only participants who received Pfizer BNT162b2 were considered eligible. Samples were de-identified using an anonymous code assigned to each sample. Only samples without bloodborne pathogens, including HIV, HTLV-1, HBV, and HCV, were used for subsequent experiments. One potential bias that may be present is that adult participants were recruited from healthcare workers at Kyoto University Hospital, whereas older participants were mostly recruited from the general population. As healthcare workers are generally more careful about their health but also tend to be exposed to various pathogens at work, their immune response may not reflect that of the general adult citizens. As older adults who participated in this study were recruited openly by the internet, they might be more health-conscious and therefore report more adverse events than the general older population.
Ethics oversight	This longitudinal study was reviewed and approved by the Kyoto University Graduate School and Faculty of Medicine, Ethics Committee (R0418).

Note that full information on the approval of the study protocol must also be provided in the manuscript.

Field-specific reporting

Please select the one below that is the best fit for your research. If you are not sure, read the appropriate sections before making your selection.

☒ Life sciences ☐ Behavioural & social sciences ☐ Ecological, evolutionary & environmental sciences

For a reference copy of the document with all sections, see nature.com/documents/nr-reporting-summary-flat.pdf

Life sciences study design

All studies must disclose on these points even when the disclosure is negative.

Sample size	Two hundred and twenty-five participants applied to participate in the study. No statistical methods were used to pre-determine sample sizes but our sample sizes are similar to those reported in previous publications (ref 16, 57)
Data exclusions	All donors were otherwise healthy and did not report any ongoing severe medical conditions, including cancer, gastrointestinal, liver, kidney, cardiovascular, hematologic, or endocrine diseases. Participants taking medications that may affect the immune system, including steroids or immunomodulatory drugs, were excluded. For the first and second doses, only participants who received Pfizer BNT162T2 were considered eligible. Six did not meet the eligibility criteria, and a total of 219 individuals consisting of 107 adults (aged less than 65 years, mainly workers at Kyoto University Hospital) and 112 older individuals (aged more than 65 years, mainly healthy Japanese citizens) were enrolled in the study. Two patients were lost during follow-up, and one was removed because mRNA-1273 injections were used for primary and booster vaccination (Extended Data Fig. 1b).
Replication	Samples for each patient were analyzed once due to limited availability.
Randomization	As this was an observational study, randomization was not applied.
Blinding	The investigators were not blinded to allocation during this study and outcome assessment.

Reporting for specific materials, systems and methods

We require information from authors about some types of materials, experimental systems and methods used in many studies. Here, indicate whether each material, system or method listed is relevant to your study. If you are not sure if a list item applies to your research, read the appropriate section before selecting a response.

Materials & experimental systems

n/a	Involved in the study
☐	☒ Antibodies
☒	☐ Eukaryotic cell lines
☒	☐ Palaeontology and archaeology
☒	☐ Animals and other organisms
☒	☐ Clinical data
☒	☐ Dual use research of concern

Methods

n/a	Involved in the study
☒	☐ ChIP-seq
☐	☒ Flow cytometry
☒	☐ MRI-based neuroimaging

Antibodies

Antibodies used

Product name (manufacturer, catalogue number, clone, dilution)

Brilliant Violet 421™ anti-human CD279 (PD-1) Antibody (Biolegend, 329920, EH12.2H7, 1:100)
Brilliant Violet 510™ anti-human CD57 Recombinant Antibody (Biolegend, 393314, QA17A04, 1:2500)
Brilliant Violet 570™ anti-human CD8a Antibody (Biolegend, 301038, RPA-T8, 1:500)
Brilliant Violet 605™ anti-human CD154 Antibody (Biolegend, 310826, 24-31, 1:100)
Brilliant Violet 650™ anti-human CD69 Antibody (Biolegend, 310934, FN50, 1:100)
Brilliant Violet 750™ anti-human CD28 Antibody (Biolegend, 302970, CD28.2, 1:50)
Brilliant Violet 785™ anti-human CD95 (Fas) Antibody (Biolegend, 305646, DX2, 1:200)
FITC anti-human CD196 (CCR6) Antibody (Biolegend, 353412, G034E3, 1:100)
CD4 Monoclonal Antibody (RPA-T4), Alexa Fluor™ 532 (Invitrogen, 58-0049-42, RPA-T4, 1:100)
PE anti-human CD185 (CXCR5) Antibody (Biolegend, 356904, J252D4, 1:100)
PerCP/Cyanine5.5 anti-human CD45RA Antibody (Biolegend, 304122, HI100, 1:100)
CD3 Monoclonal Antibody (OKT3), PerCP-eFluor™ 710 (Invitrogen, 46-0037-42, OKT3, 1:250)
PE/Cyanine7 anti-human CD137 (4-1BB) Antibody (Biolegend, 309818, 4B4-1, 1:100)
APC anti-human CD183 (CXCR3) Antibody (Biolegend, 353708, G025H7, 1:50)
APC/Cyanine7 anti-human CD197 (CCR7) Antibody (Biolegend, 353212, G043H7, 1:100)
Brilliant Violet 421™ anti-human Perforin Antibody (Biolegend, 308122, dG9, 1:100)
Brilliant Violet 605™ anti-human CD45RA Antibody (Biolegend, 304134, HI100, 1:100)
Brilliant Violet 650™ anti-human TNF-α Antibody (Biolegend, 502938, MAb11, 1:100)
FITC anti-human CD197 (CCR7) Antibody (Biolegend, 353216, G043H7, 1:100)
PE anti-human IL-4 Antibody (Biolegend, 500810, MP4-25D2, 1:100)
PerCP/Cyanine5.5 anti-human/mouse Granzyme B Recombinant Antibody (Biolegend, 396412, QA18A28, 1:100)
PE/Cyanine7 anti-human IL-2 Antibody (Biolegend, 500326, MQ1-17H12, 1:100)
APC anti-human IFN-γ Antibody (Biolegend, 502512, 4S.B3, 1:100)
APC/Cyanine7 anti-human IL-17A Antibody (Biolegend, 512320, BL168, 1:100)

Validation

All antibodies used in this study are commercially available and were validated by their manufacturers or in the manuscript. Information is accessible on the manufacturer's website with catalog numbers. We define the optimal titers for positive/negative separation by serial dilution.
The websites of the manufactures:
https://www.biolegend.com/ja-jp
https://www.thermofisher.com/jp/ja/home.html

Flow Cytometry

Plots

Confirm that:

☒ The axis labels state the marker and fluorochrome used (e.g. CD4-FITC).

☒ The axis scales are clearly visible. Include numbers along axes only for bottom left plot of group (a 'group' is an analysis of identical markers).

☒ All plots are contour plots with outliers or pseudocolor plots.

☒ A numerical value for number of cells or percentage (with statistics) is provided.

Methodology

Sample preparation

Whole blood was drawn into Vacutainer CPTTM Cell Preparation Tubes with sodium citrate (BD biosciences), according to the manufacturer's instructions, and processed within 2 hours to isolate peripheral blood mononuclear cells (PBMCs). Isolated PBMCs were resuspended in CELLBANKER 1 (ZENOGEN PHARMA) at a concentration of 8×10^6 cells/mL and aliquoted in 250 or 500 ml per cryotube. Samples were stored at -80 ℃ on the day of collection and in liquid nitrogen until used for the assays. Cryopreserved PBMCs were thawed in pre-warmed X-VIVO15 (LONZA) without serum. After centrifugation, the cells were washed once and used directly for assays.

Instrument

NL-3000 (Cytek)

Software	SpectroFlo software v2.2 (Cytek), FlowJo software version 10.8.1
Cell population abundance	As we used all the cells after AIM and ICS assays for phenotypic analysis, sorting was not performed in this study.
Gating strategy	The detailed gating strategies for individual markers are described in Extended Data Fig. 2 and 3. The subset definitions and gating strategies are outlined in the text or figure legends.

☒ Tick this box to confirm that a figure exemplifying the gating strategy is provided in the Supplementary Information.

March 2021
